# Analysis of biosurfactants from industrially viable *Pseudomonas* strain isolated from crude oil suggests how rhamnolipids congeners affect emulsification property and antimicrobial activity

**DOI:** 10.3389/fmicb.2014.00696

**Published:** 2014-12-22

**Authors:** Palashpriya Das, Xin-Ping Yang, Luyan Z. Ma

**Affiliations:** ^1^Biofilm Research Group, State Key Laboratory of Microbial Resources, Institute of Microbiology, Chinese Academy of SciencesBeijing, China; ^2^Institute of Microbiology, Xinjiang Academy of Agricultural SciencesUrumqi, China

**Keywords:** biosurfactant, rhamnolipid congeners, emulsification, thin layer chromatography, MRL-DRL proportion, antimicrobial action, biofilm disruption

## Abstract

Rhamnolipid biosurfactants produced mainly by *Pseudomonas* sp. had been reported to possess a wide range of potential industrial application. These biosurfactants are produced as monorhamnolipid (MRL) and di-rhamnolipid (DRL) congeners. The present study deals with rhamnolipid biosurfactants produced by three bacterial isolates from crude oil. Biosurfactants produced by one of the strains (named as IMP67) was found to be very efficacious based on its critical micelle concentration value and hydrocarbon emulsification property. Strikingly, antimicrobial, and anti-biofilm potential of this biosurfactant were higher than biosurfactants produced by other two strains. Thin layer chromatography analysis and rhamnose quantification showed that the rhamnolipids of IMP67 had more MRL congeners than biosurfactants of the other two strains. Emulsification and antimicrobial actions were affected by manual change of MRL and DRL congener proportions. Increase of MRL proportion enhanced emulsification index and antimicrobial property to Gram negative bacteria. This result indicated that the ratio of MRL and DRL affected the emulsification potentials of rhamnolipids, and suggested that high emulsification potentials might enhance rhamnolipids to penetrate the cell wall of Gram negative bacteria. In line with this finding, rhamnolipids of IMP67 also reduced the MIC of some antibiotics against bacteria, suggesting their synergistic role with the antibiotics.

## INTRODUCTION

Amphipathic surface-active agents of biological origin are referred to as biosurfactants and they significantly reduce the surface tension of water from a value of 72 mN m^-1^ to as low as 22 mN m^-1^ (Mukherjee et al., 2006). They have affinity for both polar and non-polar media. Their several advantages over their chemical counterparts include mild production conditions from inexpensive substances (Mukherjee et al., 2006), lower toxicity (Poremba et al., 1991), better environmental compatibility and biodegradability (Zajic et al., 1977; Georgiou et al., 1990). They possess the property of retaining their activity at extremes of temperature, pH and salt concentration (Banat, 1995). They exhibit antibacterial, antifungal, and antiviral properties as well as anti-adhesive action against several pathogenic microorganisms (Cameotra and Makkar, 2004; Singh and Cameotra, 2004; Rodrigues et al., 2006; Das et al., 2009a). They also find application in enhanced oil recovery (Gautam and Tyagi, 2006) and bioremediation (Singh and Cameotra, 2004; Das et al., 2009b).

Different bacteria produce different classes of biosurfactants which serve varied purposes in the producer strains (Desai and Banat, 1997). Rhamnolipids are glycolipids biosurfactants produced by *Pseudomonas aeruginosa*, which emulsify oil and reduce the surface tension of water from 72 mN m^-1^ to around 25–30 mN m^-1^ (Itoh et al., 1971; Parra et al., 1989). They find applications in tertiary petroleum recovery (Parra et al., 1989), decontamination of marine oil pollution, soil remediation (Banat, 1995; Lotfabad et al., 2009) and crop protection (Stanghellini and Miller, 1997). They also show antimicrobial activity against Gram-positive and Gram-negative bacteria probably by interaction with the phosphatidylethanolamine moiety of biological membrane systems (Sanchez et al., 2006; Stipcevic et al., 2006). Rhamnolipid also affects the biofilm architecture in *P. aeruginosa* (Davey et al., 2003). Rhamnolipid production had been reported to start soon after inoculation and most of it was produced as a secondary metabolite, i.e., the production was under control of quorum sensing system and occurred after bacterial growth ceased (Haba et al., 2003). They are produced as homologues mainly rhamnosyl-β-hydroxydecanoyl-β-hydroxydecanoate [monorhamnolipid (MRL)] and rhamnosyl-rhamnosyl-β-hydroxydecanoyl-β-hydroxydecanoate [di-rhamnolipid (DRL; Ochsner and Reiser, 1995; Abdel-Mawgoud et al., 2010)]. MRL are precursors of DRL. Generally more DRLs are produced (Deziel et al., 1999) but predominance of MRLs had also been reported (Sim et al., 1997; Costa et al., 2006). Predominance of rhamnolipid congeners depends on the bacterial strain used, carbon substrate, age of culture and culture conditions (Bharali and Konwar, 2011). The ratio of MRL to DRL is strain-dependent and changes during bacterial cultivation (Muller et al., 2011).

The present work reports the antimicrobial potentials and biofilm disruption potentials of rhamnolipid biosurfactant produced by three *Pseudomonas* strains isolated from crude oil. Under same culture conditions, one of the strains produced MRL and DRL congeners in almost equal proportion at a given point of time. The rhamnolipids of this strain also show the best antimicrobial potentials and emulsification property, while compared with the other strains that produced more DRL than MRL. The results suggested that the ratio of rhamnolipid congeners had significant contribution in the bioactivity profile. Multidrug resistance is now a worldwide problem. There is urgent need for novel antibacterial drugs or inhibitors and the present study suggested that the rhamnolipid biosurfactants could act synergistically with certain antibiotics.

## MATERIALS AND METHODS

### MICROBIAL CULTURE CONDITIONS AND THEIR MOLECULAR CHARACTERIZATION

The three rhamnolipid-producing strains used in the present work were isolated from the crude oil of Karamay W#8805, XinJiang province, China. They were designated as IMP66, IMP67 and IMP68 respectively. Luria Bertanni (LB) medium was used for the preparation of the primary inoculum. The inoculum from LB was then transferred to PPGAS medium (Gunther et al., 2005) prepared with glycerol as the carbon source for biosurfactant production. The biosurfactant production medium was also prepared with olive oil and coconut oil as carbon sources. All cultures were incubated for a week at 37∘C with an agitation speed of 200 rpm. An uninoculated medium was also incubated as a sterility control in each case. *P. aeruginosa* PAO1, known to be a rhamnolipid biosurfactant producer was grown as a positive control. DNA extraction was done from the bacterial cultures using Promega Wizard Genomic DNA purification kit (Promega, Madison, WI, USA) as per the manufacturer’s instructions. PCR amplification of the 16S ribosomal RNA gene was done with bacterial universal primers 27F and 1592R using a 35-cycle PCR (initial denaturation, 95∘C for 5 min; subsequent denaturation, 95∘C for 30 s; annealing temperature, 50∘C for 1 min; extension temperature, 72∘C for 1 min and final extension, 72∘C for 5 min). PCR amplification products were analyzed by electrophoresis on 1% agarose gel. DNA sequencing was performed at Huada, Beijing, China and nucleotide sequence similarity searches were conducted by Genbank nucleotide collection BLAST.

### STUDIES ON BACTERIAL GROWTH, BIOSURFACTANT PRODUCTION AND PIGMENT PRODUCTION

Fermentation broth samples were collected twice daily and checked for OD_600nm_, surface tension and biosurfactant concentration. Biomass was estimated by the dry weight and also by the optical density of the fermentation broth at 600 nm measured with a UV-Visible spectrophotometer (Eppendorf, Germany). The surface tension of the cell free supernatants was measured with a digital surface tensiometer (Kruss K 100, Germany) working on the principles of Wilhelmy plate method. The validity of the surface tension readings was checked with pure water (72.2 ± 0.02) before each reading. Pyocyanin pigment production by the test strains was quantified by multiplying the optical density of the acidified culture supernatant at 520 nm with 17.072 (Raoof and Latif, 2010).

### BIOSURFACTANT RECOVERY AND DETERMINATION OF CRITICAL MICELLE CONCENTRATION (CMC)

Biosurfactant was isolated from the culture broth obtained after the completion of each fermentation cycle by the standard technique (Das et al., 2008). Briefly, the fermentation broth was acidified and kept at 4∘C overnight for complete precipitation of the biosurfactant. The precipitate was then centrifuged to get the crude biosurfactants as a pellet. Following solvent extraction, rhamnolipid concentration was quantified by the orcinol-sulphuric acid method with rhamnose as the standard (Arutchelvi et al., 2011).

The critical micelle concentration (CMC) of the biosurfactants from all the media was determined by taking 50 ml of water in a round bottomed vessel and adding weighed amounts of the biosurfactant gradually to the water. The surface tension was noted every time after mixing the biosurfactant with the water by stirring. This biosurfactant addition was continued and change in surface tension was noted until it reached a constant value. Finally the first concentration at which the surface tension became constant was determined as CMC. The test was repeated and the results were expressed as an average of three independent tests.

### ASSAY OF EMULSIFICATION AND STABILITY STUDIES

The biosurfactants obtained from PPGAS cultures of the strains were checked for their ability to emulsify petrol and diesel. Equal volumes of aqueous biosurfactant solution (1 mg ml^-1^) and hydrocarbons were mixed by vortex at high speed for 5 min. The resulting mixture was incubated at 25∘C for 24 h and then the emulsification index (EI) value was calculated using the formula:

EI = (Height of emulsion layer/Height of the total mixture)×100

The emulsification of petrol and diesel by chemical surfactants like SDS and Tween 20 was also observed as a positive control.

Biosurfactant solutions were made at their respective CMCs. The solutions were heated for around 5 min to varied temperatures like 60, 70, 80, 90, and 100∘C on a block heater. The surface tension of the biosurfactant was then checked after cooling them to room temperature. To monitor the effect of pH, biosurfactant solutions at CMC were made in separate tubes and the pH values were individually adjusted from 2.0 to 10.0 by HCl or NaOH respectively. The surface tension of the resultant solution in each of the test tubes was then checked. All the tests were repeated and results were expressed as an average of three independent tests.

### DETECTION OF THE CHEMICAL NATURE OF THE BIOSURFACTANTS

Thin layer chromatography (TLC) was used to analyze the solvent extracted biosurfactants. The extracts were spotted on to 10 cm × 10 cm pre-coated silica gel TLC plates. Standard rhamnolipid was also spotted along with the samples. The solvent system used for the separation and analysis of the biosurfactants comprised of chloroform, methanol and acetic acid respectively, in a ratio of 65:15:2 (Wittgens et al., 2011). The developing jars were saturated with the solvent system for half an hour prior to the development. After development the plates were drained with detection agent consisting of 0.15 g orcinol, 8.2 ml 60% sulphuric acid and 42 ml water (Wittgens et al., 2011). The different biosurfactant spots were scrapped off from the TLC plates and suspended in a tube in a 2:1 mixture of chloroform and methanol. They were then vortexed at high speed and kept for extraction. After the extraction was complete, the contents of the tube were centrifuged to separate the silica from the solvent extracted biosurfactant. The biosurfactant extract was then transferred to fresh preweighed tubes and kept for evaporation of the solvent.The weights of the extracts were noted. Rhamnose estimation from all these extracts was then done and quantitation was done from the standard curve for detection of rhamnose.

### DETERMINATION OF ANTIMICROBIAL ACTIVITY

Agar diffusion test was used to determine the susceptibility of a few bacterial strains like *Streptococcus epidermidis*, *Bacillus subtilis*, *Staphylococcus aureus* and *Escherichia coli* to the biosurfactants. Overnight grown cultures of these bacterial strains were used to inoculate the agar plates. Biosurfactant solutions (1 mg ml^-1^) from all strains were added aseptically into cups bored on these plates. The plates were incubated at 37∘C for 24 h and zone of inhibition (ZOI) of microbial growth were measured. All tests were performed in triplicate and the inhibition zone diameter values represented the mean value ± SD.

Minimum inhibitory concentrations (MICs) of biosurfactants from all the cultures were determined by the broth microdilution assay (Das et al., 2008). Overnight grown cultures of the bacterial test strains, adjusted to a final density of 10^5^ cfu ml^-1^ were used to inoculate 96-well microtiter plates containing serially diluted biosurfactant congeners. In these tests the uninoculated growth medium served as the negative control, the positive control was the test cultures and the sterility control was the growth medium with the biosurfactant congeners. Plates were incubated at 37∘C for 24 h and then bacterial growth was monitored by absorbance at 600 nm in a microtiter plate reader. The minimum concentration of biosurfactant at which less than 50% growth of test organisms was observed, were defined as MIC_50_ values. All the tests for determination of MIC were performed in triplicate.

Minimum inhibitory concentration values of standard antibiotics like ampicillin, streptomycin, tetratcyclin, kanamycin, and tobramycin were determined against the same microbial strains used for checking antimicrobial activity of the biosurfactant. The procedure followed was the same as that mentioned earlier. Another test aimed at observing any change in MIC of antibiotics for the microbes was performed with a mixture of equal proportions of antibiotic and biosurfactant from IMP67 strain following the same procedure. MICs of the MRL and DRL congeners were also determined by following the same procedure as that for determining MICs of biosurfactants.

### ANTIADHESIVE POTENTIAL OF THE BIOSURFACTANT

Microbial adhesion inhibition activity upon surface conditioning by the biosurfactant from IMP67 strain was tested using a previously reported anti-adhesion assay (Das et al., 2009a). Briefly, 200μl of increasing concentrations of the biosurfactant (1–100μg ml^-1^) were poured in wells of a sterile 96-well flat-bottomed tissue culture polystyrene plate with lid. The plates were incubated for 18 h at 4∘C and subsequently washed twice with PBS. Bacterial test strains, against whom the antimicrobial action was tested, were cultured overnight in LB broth at 37∘C to obtain an inoculum of ∼10^5^ cells ml^-1^ (according to Mc Farland turbidity standards). An aliquot of 200 μl was added and incubated in the wells for 24 h at 4∘C. Unattached microorganisms were removed by washing the wells thrice with PBS. The remaining adherent microorganisms were fixed with 200 μl of 99% methanol per well, and after 15 min the plates were emptied and left to dry. Then the plates were stained with 200 μl of 2% crystal violet for 5 min. Excess stain was rinsed off by placing the plate under running tap water and the plates were allowed to dry. After the plates were air dried, the dye bound to the adherent microorganisms was resolubilized with 200 μl of 33% (v/v) glacial acetic acid per well. The optical density readings of each well were taken at 560 nm with a micro plate reader. The test was repeated thrice and the results expressed as the mean ± SD of three independent experiments.

Biofilm disruption activity of biosurfactant against bacteria adhering to a surface was done by a separate experiment, which was similar to the prior antiadhesive activity on biosurfactant-preconditioned surface. In this experiment, the sterile 96-well plates were incubated for a week with 200 μl inoculum of ∼10^5^ bacteria ml^-1^ (according to Mc Farland turbidity standards) of the different bacterial strains tested for antiadhesive activity at 37∘C. Control wells contained only sterile PBS. Unattached microorganisms were removed by washing the wells twice with PBS. 200 μl of the purified biosurfactant of different concentrations ranging from 1 to 100 μg ml^-1^ were added into the wells. The plates were incubated for about 24 h. The plates were washed thrice and the adherent population was fixed with 200 μl of 99% methanol per well. After 15 min the plates were emptied and left to dry. Then the plates were stained with 200 μl of 2% crystal violet per well for 5 min. Excess stain was then rinsed off by placing the plate under running tap water and the plates were kept to dry. After the plates were air dried, the dye bound to the adherent microorganisms was resolubilized with 200 μl of 33% (v/v) glacial acetic acid per well. The optical density readings of each well were taken at 560 nm with a micro plate reader. The test was repeated thrice and the results expressed as the mean ± SD of three independent experiments.

## RESULTS

### IDENTIFICATION OF STRAINS AND CHARACTERIZATION OF THEIR BIOSURFACTANT PRODUCTION

The bacterial strains, designated as IMP66, IMP67 and IMP68, were identified as *Pseudomonas* sp. pyr 41 (gene bank accession number:GU951459.1), *P. aeruginosa* LCD12 (GBAN:FJ194519.1) and *P. aeruginosa* D2 (GBAN:JN995663.1) respectively by 16S rRNA gene sequence analysis. Bacterial growth occurred in all the media with glycerol as the carbon substrate but no growth to very poor growth was observed with olive oil or coconut oil as the sole carbon substrate. The observed preference for glycerol to oily substrates for biosurfactant production might be because of variation in preference of carbon source from strain to strain (Sandoval et al., 2001) although hydrocarbons and vegetable oils have been reported to be good sources of rhamnolipid production by *P. aeruginosa* strains (Maier and Soberon-Chavez, 2000; Perfumo et al., 2006).

Biosurfactant production initiated at different times in the cultures of different strains. Continuous stable foaming was the qualitative indicator of biosurfactant production. IMP66 and IMP67 strains produced biosurfactant after about 24 h of inoculation while biosurfactant production commenced in the culture of IMP68 after around 48 h of inoculation. The dry weights of the biosurfactants produced by different strains were measured. Although the IMP66 strain grew faster than IMP67 strain which in turn grew faster than IMP68 strain, the amount of biosurfactant produced was in the order IMP68 > IMP67 > IMP66 (**Table 1**). The biomass obtained in case of IMP67 culture was least in comparison to other test strains which suggested that the carbon flux might be mainly directed toward biosurfactant production. The pigment production through determination of the concentration of pyocyanin indicated that IMP68 strain was a hyperproducer of pyocyanin with respect to PAO1. IMP68 produced more than 40μg ml^-1^ pyocyanin. The other two strains although found to be closely related to *P. aeruginosa*, did not produce much pyocyanin.

**Table 1:** Physicochemical properties of biosurfactants from bacteria under study.

Bacterial strains	Surface tension values (mN m^-1^)	Rhamnolipid content (g l^-1^)	CMC values (mg l^-1^)	EI values of petrol	EI values of diesel
IMP66	31.93 ± 0.04	0.8 ± 0.02	100	50	60
IMP67	29.85 ± 0.07	3.8 ± 0.1	50	70	80
IMP68	31.27 ± 0.06	4.2 ± 0.04	80	60	65
*Pseudomonas aeruginosa* PAO1	31.23 ± 0.07	2.8 ± 0.04	60	65	70

### SURFACTANTS FROM THREE STRAINS DIFFER IN THEIR CONGENER PROPORTIONS

**FIGURE 1:**
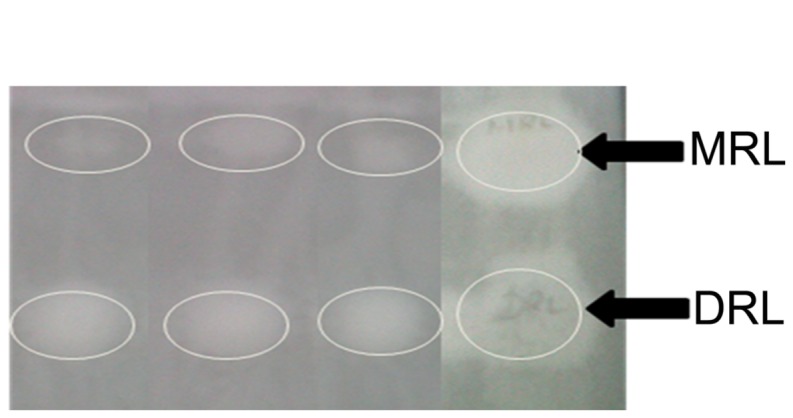
**Thin layer chromatography indicating the rhamnolipid nature of biosurfactants produced by the bacterial cultures under study.** The numbers in parentheses indicate ratios of monorhamnolipid (MRL) and di-rhamnolipid (DRL) congener proportions. The white circles indicate the area with MRL or DRL. *R*_f_ for MRL and DRL are 0.9 and 0.6 respectively.

Thin layer chromatography detected all the biosurfactants to be rhamnolipid in nature (**Figure 1**). The spots at an *R*_f_ value of around 0.9 were that of MRLs while those obtained at *R*_f_ value of approximately 0.6 were that of DRLs (Guo et al., 2009). Extraction of MRL and DRL congeners of the biosurfactant from IMP67 after scrapping off from TLC plates showed that they were almost equal in weight. A similar result was given by rhamnose quantification (Arutchelvi et al., 2011) of TLC plate scrapings of MRL and DRL fractions. In other two strains, IMP66 and IMP68, DRL was more in proportion than corresponding MRL (**Figure 1**).

### BIOSURFACTANTS OF IMP67 STRAIN ENDOWED WITH BEST PROPERTIES

The surface tension values of the cell free supernatants from cultures of all the three strains in the biosurfactant production medium recorded a surface tension value in the range of 29–31 mN m^-1^ (**Table 1**). Relative quantities of the biosurfactants produced by different strains were also determined and compared with *P. aeruginosa* PAO1 (**Table 1**). The CMC value of the biosurfactant produced by IMP67 strain was the least (**Table 1**). The lesser the value of CMC of a biosurfactant, the more is its activity which was also reflected in the emulsification potentials of this biosurfactant.

Petrol and diesel were emulsified by the IMP67 biosurfactant to high extents in comparison to the other biosurfactants (**Table 1**). This biosurfactant sustained heating upto 100∘C as its solution did not show any change in surface tension even after heating at this temperature. Biosurfactants from other two strains were stable upto 80∘C beyond which there was slight increase in the surface tension of their solutions. However, all the biosurfactants were stable toward changes of pH in the alkaline range but the surface tension of their solutions increased at around pH 2 because of biosurfactant precipitation. These results indicated that the biosurfactants from IMP67 strain can have great potential application in emulsification and crude oil recovery.

### RHAMNOLIPIDS FROM IMP67 REVEALED BEST ANTIMICROBIAL AND ANTIADHESIVE ACTIVITY

**FIGURE 2:**
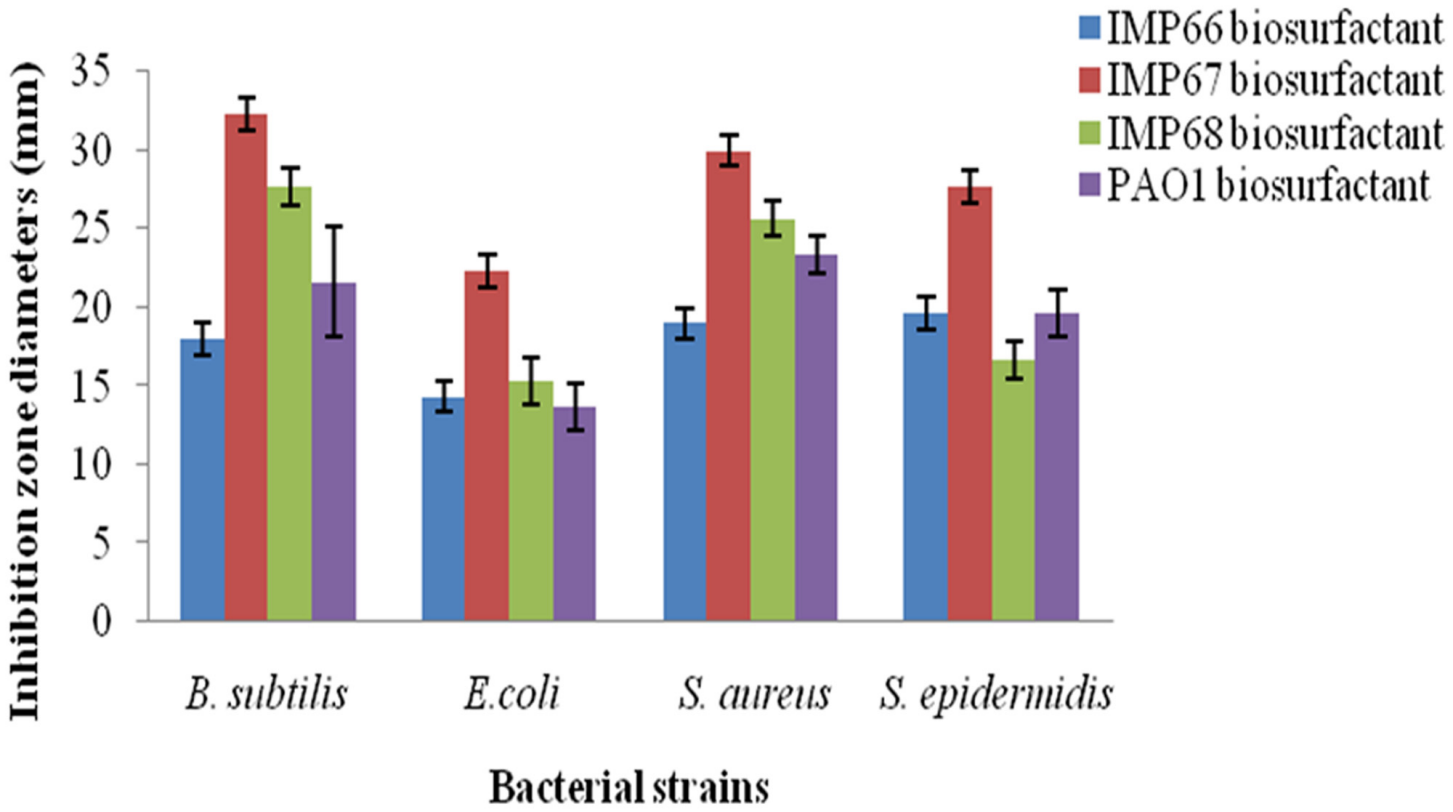
**Bigger antimicrobial inhibition zone diameters obtained with IMP67 biosurfactant in comparison to other biosurfactants**.

The rhamnolipid biosurfactants produced by IMP67 strain inhibited both Gram positive and Gram negative bacterial strains tested, i.e., *E. coli, B. subtilis, S. aureus,* and *S. epidermidis*. It produced bigger ZOI in comparison to biosurfactants from other two strains as well as *P. aeruginosa* PAO1 (**Figure 2**). The MIC of this biosurfactant against the Gram positive and Gram negative bacterial strains was least when compared to biosurfactant from IMP66, IMP68, and PAO1 strains which proved its efficiency (**Table 2**).

**Table 2:** Minimum inhibitory concentrations (MICs) of the biosurfactants.

Test bacterial strains	MICs of biosurfactants (μg ml^-1^)			
	IMP66	IMP67	IMP68	*P. aeruginosa* PAO1
*Bacillus subtilis*	16	4	6	10
*Escherichia coli*	32	4	16	30
*Staphylococcus aureus*	30	16	16	25
*Streptococcus epidermidis*	16	4	6	20

The antiadhesive activity of the biosurfactant was tested against a panel of microorganisms. The activity was found to depend on both the concentration of biosurfactant and the microorganism tested. Anti-adhesion achieved by biosurfactant conditioning of surfaces was in the range of 50–80% at a concentration of 8–64 μg ml^-1^ (**Table 3**). Biofilm dislodging was also observed significantly (**Table 3**).

**Table 3:** Anti-adhesive property of the IMP67 biosurfactant.

Test microorganisms	50% effective concentrations (EC_50_) of rhamnolipids used for surface conditioning to promote anti-adhesion (μg/ml)	50% effective concentrations (EC_50_) of rhamnolipids for biofilm disruption (μg/ml)
*B. subtilis*	8	64
*E. coli*	16	128
*S. aureus*	64	128
*S. epidermidis*	8	32

### INCREASE OF MRL PROPORTION ENHANCED EMULSIFICATION POTENTIALS AND ANTIMICROBIAL PROPERTY OF RHAMNOLIPID BIOSURFACTANT

The IMP67 biosurfactant contained more MRL than that of the other strains. The biosurfactant of IMP67 also exhibited the best emulsification potentials and antimicrobial activity. Thus we hypothesized that there was a correlation between its bioactivities and proportion of congeners. The biosurfactant congeners extracted from thin layer chromatograms were mixed in various proportions and emulsification of hydrocarbons was conducted with them in order to note whether they promote any change in emulsification. The EI values obtained by varying the proportions of the congeners showed that when the proportion of the di-congener increased with respect to the mono-congener, some decrease occurred in the EI values (**Table 4**). In contrast, slight increase in emulsification indices was observed when the di-congener proportion increased with respect to the mono-congener (**Table 4**).

**Table 4:** Changes in emulsification index and ZOIs by varying proportions of congeners from IMP67.

Varied IMP67 biosurfactant congener proportions		Changes in emulsification index		Changes in antimicrobial ZOIs	
		Petrol	Diesel	Gram positive bacterium (*B. subtilis*)	Gram negative bacterium (*E. coli*)
MRL:DRL	1:1	70	80	33.0 ± 0.0	20.0 ± 0.0
	1:3	60	75	32.0 ± 0.0	18.66 ± 0.57
	1:5	50	60	30.0 ± 0.0	15.66 ± 0.57
	3:1	75	85	32.0 ± 0.0	27.33 ± 0.57
	5:1	80	90	30.0 ± 0.0	30.0 ± 0.0
	6:0	70	80	28.33 ± 0.57	30.0 ± 0.0
	0:6	50	50	25.66 ± 0.57	12.0 ± 0.0

These different congener mixtures were also checked for their antimicrobial action and the variation in congener proportion varied the diameter of ZOI markedly (**Table 4**). The change in antimicrobial ZOI against the Gram negative bacterium tested was more prominent than that in the Gram positive one. The ZOI in Gram negative bacterium increased when the MRL was more in proportion than the DRL. Momo- to di-RL ratio in 5:1 exhibited the greatest EI values and the best antimicrobial activity against both Gram positive and negative bacteria. MRL congener alone showed bigger inhibition zone against bacteria and better emulsification activity than that of DRL (**Table 4**), although MIC analysis showed MRL and DRL congeners of IMP67 biosurfactant had similar MIC values against most of strains tested (**Table 5**). These results indicated that increase of MRL proportion enhanced antimicrobial activity of rhamnolipids from IMP67.

**Table 5:** Minimum inhibitory concentration values of individual congeners from IMP67.

Strains	*B. subtilis*	*E. coli*	*S. aureus*	*S. epidermidis*
MIC of MRLs (μg ml^-1^)	4	4	16	4
MIC of DRLs (μg ml^-1^)	2	4	16	4

### THE POTENTIAL OF RHAMNOLIPIDS BIOSURFACTANT TO ACT SYNERGISTICALLY WITH ANTIBIOTICS

Antimicrobial activity of biosurfactants was proposed by interaction with biological membrane systems. Thus we hypothesized that rhamnolipids can be used synergistically with other antibiotics to help their penetration into bacteria. MIC values of standard antibiotics against the test microbial strains were determined in combination with equal proportions of the IMP67 biosurfactant and the change in the MIC values showed a varied pattern in different strains (**Table 6**). In *B. subtilis*, a huge change in the MIC of tetracycline was observed when treated along with IMP67 biosurfactant while there was no change in the MICs of ampicillin, gentamicin, and kanamycin in conjugation with biosurfactant. When the test bacterium was *E. coli*, there was no change in MIC of gentamicin and streptomycin in collaboration with biosurfactant while all the other antibiotics got their MICs reduced (**Table 6**). MICs of ampicillin and kanamycin against both *S. aureus* and *S. epidermidis* reduced in presence of the biosurfactants from IMP67 while the MICs of other drugs did not change (**Table 6**). All these results suggested the efficacy of the biosurfactants as a potential drug synergist against the drug resistant bacteria.

**Table 6:** Minimum inhibitory concentrations of biosurfactant from IMP67 in conjugation with standard antibiotics.

Test bacterial strains	MICs of antibiotics (μg ml^-1^)		MICs of antibiotics with IMP67 biosurfactant (μg ml^-1^)
*B. subtilis*	Tetracyclin	25	15
	Streptomycin	4	2
*E. coli*	Ampicillin	4	2
	Tetracyclin	10	4
	Tobramycin	6	4
	Kanamycin	8	6
*S. aureus*	Ampicillin	6	2
	Kanamycin	6	4
*S. epidermidis*	Ampicillin	4	1
	Tetracyclin	10	4
	Kanamycin	4	1

## DISCUSSION

Rhamnolipid biosurfactants produced by the strains under investigation were potent hydrocarbon emulsifiers which can make them suitable candidates for promoting environmental remediation. Although the IMP67 strain was found to be a close relative of *P. aeruginosa*, it did not produce copious amounts of pyocyanin and hence devoid of deleterious effects of this pigment. Most importantly, this strain is also a good biosurfactants producer, making it to be an industrially viable strain. Antimicrobial potentials of the rhamnolipid biosurfactants are well documented (Bharali and Konwar, 2011) but the antimicrobial potential of MRL and DRL congeners separately have not been tapped earlier to the best of our knowledge. The low MIC values of the IMP67 biosurfactant, as a whole, as well as its congeners separately, against the test bacterial strains in comparison to earlier reported ones (Bharali and Konwar, 2011) also advocate for the potency of these compounds. The higher antimicrobial potential of this biosurfactant is thought to be because of the equal congener proportions. As the MRLs and DRLs were present in almost similar amounts, there may be some sort of synergistic action on the overall antimicrobial property of the biosurfactant thus leading to the lesser MIC values of this biosurfactant against the strains tested. Reports on microbial production of varied biosurfactant structures under different growth conditions are there and structures were reported to promote variation in their action potentials. In the present work, detailed structural investigation needs to be done to get some insight in this direction. However, the degree of hydrophobicity also plays a role in exhibiting antimicrobial action. In this work, as the proportion of mono- to di-changed in the order of 1:1, 1:2 and so on, there was an enhancement in the polar nature of the compound and this resulted in the decrease of its antimicrobial action. On the other hand, when the proportion of di- to mono-rhamnolipid changed in the same manner, antimicrobial inhibition zone diameters increased, especially in case of the Gram negative bacteria. This enhancement may be accounted for by the increment in hydrophobic nature of the mixture which supposedly favored its increased penetration through lipids of the bacterial cell wall. Interestingly, DRL alone showed a decreased emulsification potential than the whole biosurfactant. Although it was reported that DRL was more useful in bioremediation (Zhang et al., 1997) but in this work in the IMP67 biosurfactant, MRL seemed to play a significant role in emulsification and antimicrobial potentials.

Anti-adhesion potential of IMP67 biosurfactant was also worth mentioning. Biosurfactant-conditioning of any surface, to which the bacteria might attach, inhibits bacterial adhesion in contrast to inhibition of bacterial adhesion either by exclusion or steric hindrance mechanism (Zá rate and Nader-Macias, 2006). In the present study, adhesion inhibition mediated by both surface conditioning and biosurfactant-mediated biofilm dislodging was observed which may find potential applications in protection of the surfaces of surgical instruments where no microbial load is desirable.

## Conflict of Interest Statement

The Review Editor Jun-Jie Zhang, declares that, despite being affiliated to the same institution as authors Palashpriya Das and Luyan Z. Ma, the review process was handled objectively and no conflict of interest exists. The authors declare that they have filed two provisional patent applications on the industrially use of strain IMP67 and IMP68 (PRC Patent Application No. 201310135130.1 and 201310135091.5 respectively). This does not alter our adherence to all policies on sharing data and materials.
